# The SARS-CoV-2 pandemic as a source of unprecedented bioethical and biolaw issues: lessons for intensivists

**DOI:** 10.1186/s44158-023-00092-9

**Published:** 2023-04-17

**Authors:** Davide Mazzon, Mariassunta Piccinni, Anna Aprile, Alberto Grassetto, Silvia De Rosa, Fabio Baratto, Silvio Marafon, Fabio Toffoletto, Paolo Rosi, Paolo Navalesi

**Affiliations:** 1 Anesthesia and Intensive Care, Belluno Hospital, Veneto Region ICU Network, Belluno, Italy; 2 Department of Political Sciences, Law and International Studies, University of Padua, Padova, Italy; 3 Department of Cardiac, Thoracic, Vascular Sciences and Public Health, University of Padua, Padova, Italy; 4 Anesthesia and Intensive Care, Vittorio Veneto Hospital, Veneto Region ICU Network, Via C. Forlanini 71, Vittorio Veneto, Treviso 31029 Italy; 5 Centre for Medical Sciences - CISMed, University of Trento, Anesthesia and Intensive Care, Santa Chiara Regional Hospital, Veneto Region ICU Network, APSS Trento, Trento, Italy; 6 Anesthesia and Intensive Care, Ospedali Riuniti Padova Sud, Veneto Region ICU Network, Monselice, Italy; 7 Anesthesia and Intensive Care, Arzignano Hospital, Veneto Region ICU Network, Arzignano, Italy; 8 Anesthesia and Intensive Care, San Donà and Portogruaro Hospitals, Veneto Region ICU Network, San Donà di Piave, Italy; 9 Department of Emergency Medical Service System, Veneto Region ICU Network, Venezia, Veneto Region Italy; 10 Depatment of Medicine - DIMED - University of Padua, Institute of Anesthesia and Intensive Care, Padua University Hospital, Veneto Region ICU Network, Padova, Italy

**Keywords:** Covid-19, Pandemic, Public health emergency, Adult palliative care, Informed consent

## Abstract

The following article presents the relevant and unprecedented bioethical and biolaw issues posed by the SARS-COV-2 pandemic and summarizes the initiatives adopted by the Italian Society of Anesthesia and Resuscitation (SIAARTI) as well as by the Veneto Region ICU Network. Since the initial phase of the pandemic, in March 2020, there has been a strong appeal from both SIAARTI and the Veneto Region ICU Network to consider “the appropriate intensive treatment.” During the pandemic, the principle of proportionality must be applied, in compliance with the main principle in bioethics. This encompasses the concept of clinical appropriateness, based on the efficacy of the treatment in specific case and context, as well as the concept of ethical appropriateness, which refers to ethical and juridical principles of acceptance of health care. The “appropriate treatment” must never interfere with the withdrawal of patients, who are not eligible for intensive treatments since they would not benefit from them and who are eligible for ordinary treatments that must be maintained, and, where necessary, palliative treatments were initiated. On the other hand, it must not encroach on unreasonable obstinacy. At the end of 2020, the SIAARTI-SIMLA (Italian Society of Insurance and Legal Medicine) document provides healthcare professionals with a tool for responding appropriately to the emergency of the pandemic, in the event of an imbalance between healthcare demand and available resources. The document states that the ICU triage should be based on global evaluation of each patient, taking into account well-defined parameters and stresses that each person potentially eligible for intensive care should have a shared care planning (SCP) stipulated, and, when necessary, a proxy should be nominated. This has illustrated how the biolaw issues encountered by intensivists during the pandemic, such as those relating to consent and refusal to medical treatment, even when it is lifesaving, as well as requests for treatment of unproven clinical efficacy, were subject to appropriate guidelines and solutions through the application of Law 219/2017 (provisions for informed consent and advance directives treatment). Communication with family members and the management of sensitive personal data; the evaluation of “legal capacity” of comprehension and informed decision-making regarding the proposed treatment plan; and the need for emergency medical intervention in the absence of consent are all addressed in light of the relevant regulations and the particular conditions of social isolation induced by the pandemic. The collaborative ICUs network sustained by the Veneto Region has given great prominence to clinical bioethics issues, and as a result, multidisciplinary integration with the help of legal and juridical experts was developed. This has led to an increase in skills in the bioethical field, as well as providing a valuable lesson for the improvement of therapeutic relationships with critically ill patients and their families.

## Introduction

The SARS-CoV-2 pandemic has overwhelmed the Italian national health system and Italian ICUs since February 2020 and has caused to date (February 20, 2023) in Veneto Region 16,226 deaths. Due to adherence to mass vaccination campaigns, registered cases subsided in just over a couple of years, yet the pandemic has posed unprecedented and relevant issues of bioethical, deontological, and biolaw nature [1]. As of February 14, 2023, and for the first time in 3 years, the counts of Covid-19-reported cases in Veneto Region have dropped below 100, which has allowed us to make a definitive assessment of the lessons learnt in this 3-year period that have highlighted bioethical issues such as the following: limited allocation of resources, compulsory vaccination, communication of scientific contents to the public, and the public’s willingness to trust in science.

Physicians, not only as doctors but also as citizens, had to face the impact of the lockdown and its significant limitations on individual freedom, such as restrictions on access to hospitals and nursing homes to reduce the risk of the spreading Covid-19, with its potential risk of dehumanizing relationships with the elderly and frail, as well as leading to difficulty in guaranteeing adequate healthcare both for patients affected by SARS-CoV-2-related diseases as well as other diseases. This can be accounted for by the disproportion between demand and resource availability. In addition, there has been (an added) difficulty with respect to treatment management due to people being distrustful not only of the efficacy of vaccines but also of the critical care available and provided for treatment of SARS-CoV-2 pneumonia, including those treated in ICUs settings. The relevant and unprecedented bioethical, deontological, and biolaw issues posed by the pandemic have been a subject of reflection, discussion, of reports, and training events in Veneto Region, thanks to the initiatives of the Veneto Region ICU Network, which this article intents to summarize.

## The pandemic context and bioethical guidelines for ICUs in Italy and in Veneto Region

In the first hectic days of the rapid spread of SARS-CoV-2 in Italy, March 2020, SIAARTI made the courageous effort to try and provide a common framework for admission of patients to intensive care treatments during the pandemic emergency, in order to increase survival rates of patients infected with SARS-CoV-2 [2].

In the document entitled “Recommendations for the allocation of intensive care treatments in exceptional resources limited circumstances” dated June 3, 2020, SIAARTI highlighted the need to apply the principles of clinical appropriateness and proportionality which are at the basis of our profession and which, moreover, should already be applied on a daily basis by anesthetists. Subsequently, on March 3, 2020, the Veneto Region ICU Network approved a document entitled “Ethical decision making for treatment of patients suffering from severe respiratory failure secondary to Covid-19 infection” [3]. This document was sent by the General Directorate of Health and Social Care to all the general directorates of the local health units and hospitals of the Veneto Region, subsequent to the aforementioned SIAARTI document [2]. The following document alludes to the previous one and affirms that homogenous implementation in all hospital structures should be applied and in particular in the contexts of high intensity care, in order to avoid different assistance and decision-making strategies in the region.

The Veneto Region document underlines how in the pandemic context “the anesthesiologist who decides on intensive care accessibility, does not carry out a despotic act but accomplishes the highest and most complex form of responsibility, being held accountable for the balance between possible benefits to the patient and the risk of prolonging an agonal state.”

Since the initial phase of the pandemic, there has been a strong appeal from SIAARTI and the Veneto Region ICU Network to take “the appropriate intensive treatment” into consideration. During the pandemic, the principle of proportionality must be applied, in compliance with the main principle in bioethics. This includes the concept of clinical appropriateness, based on the efficacy of treatment in specific cases and context, taking into account both the biological age of the patient (which differs from chronological age), their functional status and concomitant comorbidities, and the concept of ethical appropriateness which refers to the ethical and juridical principle of acceptance of health care.

The “appropriate treatment” must never interfere with the withdrawal of patients, who are not eligible for intensive treatments since they would not benefit from them and who are eligible for ordinary treatments that must be maintained, and, where necessary, palliative were treatments initiated. On the other hand, it must not encroach on unreasonable obstinacy in patients who do not respond to intensive treatments and for whom Art. 2, par. 2, of Law 219/2017 states: “In cases of patients with poor short-term prognosis or imminent death, doctors must abstain from administering unnecessary or disproportionate treatment with unreasonable obstinacy.”

Bioethical and biolaw considerations established at the beginning of the pandemic were then proceeded at the end of 2020 by the SIAARTI-SIMLA document “Decisions for intensive care when there is an imbalance between care needs and resources during the Covid-19 pandemic [4].” After public consultations, the final version of the document was published on the National Centre for Clinical Excellence, quality and safety of care website on the January 13, 2021 in the section entitled “Best Clinical Practice.” The general objective of the document was to provide healthcare professionals with a tool for responding appropriately to the emergency due to the current Covid-19 pandemic in the event of an imbalance between healthcare demand and available resources, with particular reference to ICUs resources. The document states that the purpose of ICUs triage is to guarantee treatments to the greatest possible number of critically ill patients who can derive clinical benefit from them, based on prognostic parameters that are well supported by scientific evidence. The document clarifies that the evaluation of each case aims at stratifying the probabilities of overcoming critical conditions with the support of intensive care: “it should be based on global evaluation of each patient, taking into account the following parameters: number and type of comorbidities; previous functional status and frailty in response to treatment; severity of the current clinical condition; presumable impact of intensive treatments, taking into consideration the patient's age and patient’s wishes regarding intensive care, which should be inquired into as early as possible in the initial triage phase.” To avoid misunderstandings, the document specifies that age “must be considered as part of the global assessment of the patient, and in itself it is not a criterion for deciding which patients are most likely to benefit from intensive care an,d therefore, cannot be used by establishing predefined age cutoffs in the triage.” It then states that “all patients who we foresee may need future life support treatment should be offered SCP.”

It should be noted that in Veneto Region during the pandemic, no patient requiring intensive care was unable to receive it due to lack of resources. This was possible due to the application of the criteria of appropriateness for ICU admission which excluded hospitalization of patients who would not have benefited from it or whom nonetheless could have received appropriate care in a different care setting.

## The issue of consent and refusal of intensive care during the pandemic context: general aspects

Medical ethics and deontology set out certain duties: the duty to promote and protect the health of individuals and the relief of suffering that respect patients’ wishes. Intensivists have a deontological duty to treat all those who they believe can benefit from intensive treatment, respecting the principle of autonomy and self-determination of the patient and respecting the possibility of refusal to medical treatment even when it is lifesaving.

In the diagnostic and therapeutic pathways, the doctor-patient relationship has the characteristics of a “clearing house” between 2 entities: the physicians on the one hand, which increasingly involves a multidisciplinary team approach and scientific evidence, with his or her rights, which are constitutionally guaranteed and acknowledged by the Law 219/2017 (provisions for informed consent and advance directives treatment).

In general, the legal and deontological principles of any medical act, in the absence of the patient’s consent, are unlawful. The patient, therefore, always has the right to refuse medical treatment, even when it is lifesaving. Pursuant to Art. 1, par. 6, of Law 219/2017, “The doctor is obliged to respect the will expressed by the patient to refuse health treatment or to withdraw from it and, as a result, is exempt from civil or criminal liability.” There is, therefore, no doubt that once verified, specifically in the context of the SARS-CoV-2 pandemic, the refusal to undergo certain treatments, even lifesaving, must be respected. In other words, the refusal must be freely given, informed, aware of their rights, specific, unambiguous, explicit, revocable, and expressed by an individual in full possession of capacity.

Law 219/2017 provides advance directives, intended to give autonomous individuals some measure of control over their healthcare strategies even when they have lost the capacity to make their own decisions.

It seems to be highly significant that if a treatment plan involves incremental therapeutic steps in the face of a disease with a high probability of death, such as severe pneumonia from SARS-CoV-2, it is advisable from the onset (admission to the hospital) to proceed with SCP, Art. 5 of Law 219/2017, which needs to be part of medical records and which discloses the consent or refusal of the patient to the SCP plan (pharmacological treatment, noninvasive or invasive ventilation following tracheal intubation, and transfer to the ICU).

The right to self-determination, in the event of possible evolution of the disease (e.g., due to hypoxia, hypercapnia, delirium) and in which the patient tends towards a condition of incapacity, is guaranteed by Articles 4 and 5 which establish that the patient can nominate a proxy to represent him/her, while the Judicial Court can appoint as a legal representative or support administrator “his/her family member, the civil union, the cohabitant or a trusted person,” pursuant to Art. 408 of the Civil Code, in the relationship with healthcare professionals and organizations in the SCP.

Good communication with family members can be effective in respecting the wishes of the patient and in finding solutions that can best achieve his/her interests. The document “How to communicate with family members in conditions of social isolation,” dated April 2020, promptly provided indications on the necessary relational skills as well as implementing tools (e.g., telephones and video calls) which serve to deal with the unprecedented and sudden change in the modality of communication with relatives of all Covid-19 patients in different care settings due to social isolation [5]. The document also addressed the issue of management of personal data. Even in an emergency, when the patient is capable of understanding and expressing an opinion, consent for processing of personal data is always required, in addition to specific indications by family members authorized to receive medical information. With regard to maintaining confidentiality of sensitive personal data, the document states that “In terms of the confidentiality of personal data, it should also be highlighted that this right is not an absolute prerogative but must be considered in relation to its function in society.” Processing of personal data related to the current state of health is considered lawful when necessary to protect the best interest of that patient or other people, when the patient involved is physically or legally incapable of giving free and informed consent, and for reasons of public interest in the field of public health (see Article 9, par. 2, (letter c, i), EU Reg. 2016/679).

## The issue of consent and refusal to ICU admission in the pandemic context: specific aspects

Law 219/2017 essentially recognizes that consent for medical acts is not reached in a punctual moment; instead, it comes from a well-established professional relationship, based on a continuous process of communication and dialogue between the patient and his or her doctor. The informed consent, as a principle, incorporates a SCP that is built with the patient and becomes the fundamental basis of the medical care relationship Law 219/2017 generically refers to “legal capacity” (Art. 1, par. 5). It does not, however, take into consideration the different concepts of legal capacity that emerge in law (legal capacity to act, mental capacity ability to discern, and self-determination).

This generic reference made by Law 219/2017 to “legal capacity” brings to the legal consequence that any person of age who is not legally incapacitated according to a judicial measure is assumed to be able to make medical decisions. However, the attending physician still retains the duty to evaluate the actual ability of comprehension and informed decision-making, in the form of dialogue and creating a relationship with the patient that can be aided with the support of family members.

In bioethics, “capacity” is a fundamental requirement for the validity of informed consent or refusal of treatment and is different from that established by the Criminal Code (criminal imputability) and by the Civil Code (legal capacity for rights and obligations). “Capacity” in bioethics refers to the “ability to understand and make health decisions for oneself,” meaning that patients are able to (i) understand the relevant health information communicated to them in order to be actively involved in decision-making processes that concern them (regarding their current health conditions and evolution of their ongoing illness and therapeutic options), (ii) the patient must be fully aware of all the consequences arising from their decisions of consenting or refusing treatments, and (iii) the patient must communicate final decisions in a comprehensible manner and must motivate these choices on the basis of their beliefs and values.

In a 2011 study carried out by the Italian Group for evaluation of intensive care treatment (GIVITI) in 84 Italian ICUs, which included about 3000 patients, valid informed consent to ICU treatments was provided only by 14.4% of patients, while effective involvement in the ICU’s SCP was given by 8.1 patients [6]. Patient capacity was frequently found to be questionable, changeable, or fluctuating.

During the pandemic, the CoroNerve network created in the UK, in which the Scientific Societies of Neurological and Psychiatric Sciences were involved, reported that 30% of 125 patients infected with Covid-19 had neurocognitive impairment, and that neuropsychiatric disorders, such as delirium, were more common in younger age groups, while disorders of cerebrovascular origin prevailed in the elderly population [7]. A systematic review of neuropsychiatric disorders in patients with Covid-19 infection found the presence of delirium and impaired consciousness in 65% and 21%, respectively, in 58 and 82 patients admitted in the ICU [8]. The same authors agree on the fact that an altered mental state is also commonly found in patients admitted in the ICU due to sepsis unrelated to Covid-19 infection, with a prevalence in the elderly population, which is probably due to cognitive degeneration during polypharmacy administration, including sedative-analgesic medications.

In daily clinical practice, intensivists are often faced with patients with an altered mental state at the time of admission to the ICU or subsequently, and the physician is not automatically authorized to perform medical treatment on behalf of a patient who is deemed incapable of making reasoned medical decisions. Individuals possess self-determination in the health sector, and their refusal of medical treatment cannot be overridden unless demonstrated to be otherwise, i.e., their inability to make informed decisions about treatments. Furthermore, if it is confirmed that the patient’s refusal to treatment is caused by cognitive impairment or psychiatric conditions/psychotic disorders that impede decision-making capacity, the medical director involved in the patient’s care has the duty to make a formal application to a court for determination of incompetency and proceeds to the assignment of a surrogate to act on the patient’s behalf, or to inform the prosecutor, pursuant to Art. 406, par. 3, of the Civil Code.

With reference to both the pandemic emergency and normal situations, in the frequent cases in which the intensivist is asked to intervene on a patient in a coma or in any case lacking adequate capacity for self-determination and for whom there is no documentation of refusal for certain treatments, the physician can undertake a treatment intervention that is life-sustaining in emergent situations as established in Art. 1, par. 7, of Law 219/2017. “The physician and the members of the medical team must provide the necessary treatment, respecting the patient’s will, when his or her clinical conditions allows it.”

Autonomy and the right of self-determination, as Law 219/2017 and the deontological code, however, do not entitle the patient to insist on receiving a particular medical treatment regardless of the nature of the treatment and of unproven clinical efficacy.

The occurrence of such requests was conferred to some directorates of Anesthesia and Intensive Care Units of the Veneto Region and was the subject of in-depth analysis during a webinar held on February 3, 2022, entitled “Refusal of intensive care and the request for inappropriate/non-beneficial intensive care treatments in patients affected by Covid-19” which was endorsed by the Veneto Region ICU Network and sustained by the School of Public Health Foundation of the Veneto Region (https://www.fondazionessp.it/ita/formazione/area-sanitaria-socio-sanitaria-e-trapianti/rifiuto-di-cure-intensive-e-richiesta-di-trattamenti-incongrui-nel-paziente-affetto-da-covid-19).

Physicians can thus legitimately refuse a treatment requested by the patient if it is inappropriate in light of scientific knowledge available and current clinical conditions of the patient: “the patient cannot demand treatment contrary to the law, professional ethics or good clinical care practice and with regard to such requests, the doctor has no professional obligations.” (Art. 1, par. 6, Law 219/2017). This legal provision is in accordance with the Italian Code of Medical Ethics in the last version of 2014 in Art. 22: “Denial of Medical Care/Conscientious Objections” that affirm “The physician may refuse to provide professional care when the interventions requested are in contrast with his conscience or with his technical-scientific beliefs, as long as the refusal is not of serious and imminent harm to the health of the patient.”

Therefore, even in the pandemic context, only clinical practice supported by scientific evidence and appropriateness conforms with medical ethics and should be in the best interest of the patient. Hence, clinical treatments proposed by physicians, which are not evidence based, should not be taken into consideration in the best interest of the patient. Reasons justifying the clinical course proposed by the doctor as the most appropriate, in the light of the most up-to-date scientific evidence, should be communicated to the patient, particularly with regard to the current clinical condition in which the decision was taken.

## Conclusions

The professional context in which intensivists found themselves in during the emergency phase of the pandemic, alongside the need to equip themselves instruments to respond to clinical-organizational problems, such as the development of regional and extra-regional collaborative networks, has provided an extraordinary opportunity to highlight the unavoidable matter that concerns the criteria for accessibility to intensive care treatment and to the not so well-known Law 219/2017. This law has acted as an important regulatory framework for issues such as that of treatment relationship, respect for self-determination of patients, management of the end-of-life care, and up to the refusal of treatment and requests for inappropriate treatments. The collaborative agreement created by Veneto Region ICU Network and sustained by the Veneto Region has given great prominence to clinical bioethics issues, and as a result, multidisciplinary integration with legal and juridical experts was developed, which has led to an increase in skills in the bioethical field, as well as providing a valuable lesson for the improvement of therapeutic relationships. The latter can be summarized as follows:

1. In the case of chronic diseases or those characterized by an inevitable poor prognosis or in expectation of worsening clinical conditions, it is necessary to inform the patient in timely fashion that transfer to the ICU may be necessary, ensuring to provide adequate information on the type of treatment that might be undertaken, on potential clinical interventions, and on the probability of survival in his/her specific case as based on clinical records of the hospital.
2. If the patient has not been involved before in a SCP, it is necessary that, when feasible, SCP should be stipulated and full support with filling out forms offered; every patient should have the time to make a decision which will prevail even in the future when choice may be partially or wholly prevented by illness or when their decisional capacity might be lost. Written documents need to be stored with medical records; a legal representative or support administrator or a proxy should be nominated on the base of single patient’s requirements.
3. The medical team will maintain the relationship with the appointed legal representative or support administrator or the proxy even in case of induced coma.
4. The application of Law 219/2017 must be guaranteed with regard to the following:

-
  - The impossibility of accepting requests and demands for health treatments contrary to good clinical practices
  - The duty on the part of the medical team to treat pain with every means available and to provide palliative care, including deep palliative sedation when indicated
  - The duty on part of the medical team to refrain from any form of unreasonable obstinacy in prolonging agony unnecessarily in the event of poor prognosis in the short term

## Data Availability

Not applicable.

## Abbreviations

ICU: Intensive care unit
SCP: Shared care planning
